# Identifying Gaps in Knowledge and Barriers to Utilization of Hospice and End-of-Life Care Services

**DOI:** 10.1093/geroni/igaa057.220

**Published:** 2020-12-16

**Authors:** Lenard Kaye, Nicholas Silver, Carli Hutchison, David Wihry

**Affiliations:** University of Maine, Bangor, Maine, United States

## Abstract

Hospice and end-of-life (HEOL) services have traditionally been underutilized, especially in rural areas. A community assessment, performed in eastern Maine, sheds light on the extent to which public and health care professional attitudes and knowledge explain HEOL service access and utilization levels. Structured surveys were distributed in the fall of 2019 targeting community residents (18 years and older) and hospice/health care professionals. Residents (n=213) displayed significant levels of ignorance concerning the purpose and availability of HEOL services. Although a range of HEOL services were rated as highly important, residents were unable to confirm their actual availability. Overall, one in every two residents did not know if particular HEOL services existed locally (ranging from a low of 27% that were unsure whether grief support services were available to 67% unaware of the presence of a community hospice house). Hospice/health professionals (n=22) were aligned in their belief that the public did not have a clear understanding of the intent of HEOL services. Interestingly, community residents lacked equivalent measures of confidence in the HEOL knowledge levels of health care professionals. Significant barriers to the timely utilization of HEOL services included: stigma/fear, lack of education as to the purpose of these services, financial barriers to access, and scarcity of selected HEOL services. For both respondent groups, the top ranking suggestions for increasing HEOL services utilization were earlier discussions between providers and patients concerning the need for such services and increased public and professional education around death and dying.

